# Establishing a comprehensive web‐based analysis platform for *Nicotiana benthamiana* genome and transcriptome

**DOI:** 10.1111/tpj.17178

**Published:** 2024-12-03

**Authors:** Ken‐ichi Kurotani, Hideki Hirakawa, Kenta Shirasawa, Koya Tagiri, Moe Mori, Abedelaziz Ramadan, Yasunori Ichihashi, Takamasa Suzuki, Yasuhiro Tanizawa, Jiyuan An, Christopher Winefield, Peter M. Waterhouse, Kenji Miura, Yasukazu Nakamura, Sachiko Isobe, Michitaka Notaguchi

**Affiliations:** ^1^ Bioscience and Biotechnology Center Nagoya University Furo‐cho, Chikusa Nagoya 464‐8601 Japan; ^2^ Department of Frontier Research and Development Kazusa DNA Research Institute Kazusa‐kamatari Kisarazu 292‐0818 Japan; ^3^ Graduate School of Bioagricultural Science Nagoya University Furo‐cho, Chikusa Nagoya 464‐8601 Japan; ^4^ Tsukuba‐Plant Innovation Research Center University of Tsukuba 1‐1‐1 Tennoudai Tsukuba 305‐8572 Japan; ^5^ RIKEN BioResource Research Center 3‐1‐1 Takanodai Tsukuba 305‐0074 Japan; ^6^ College of Bioscience and Biotechnology Chubu University Matsumoto‐cho Kasugai 487‐8501 Japan; ^7^ Research Organization of Information and Systems National Institute of Genetics Yata Mishima 411‐8540 Japan; ^8^ Centre for Agriculture and the Bioeconomy Queensland University of Technology (QUT) Brisbane Queensland Australia; ^9^ ARC Centre of Excellence for Plant Success in Nature & Agriculture Brisbane Queensland Australia; ^10^ Department of Wine Food and Molecular Biosciences Lincoln University Lincoln New Zealand; ^11^ Department of Science Kyoto University, Kitashirakawa Oiwake‐cho Sakyo Kyoto 606‐8502 Japan

**Keywords:** *Nicotiana benthamiana*, RNA‐seq, nicotine biosynthesis, agroinfiltration, bioinformatics

## Abstract

*Nicotiana benthamiana* has long served as a crucial plant material extensively used in plant physiology research, particularly in the field of plant pathology, because of its high susceptibility to plant viruses. Additionally, it serves as a production platform to test vaccines and other valuable substances. Among its approximately 3.1 Gb genome, 57 583 genes have been annotated within a 61 Mb region. We created a comprehensive and easy‐to‐use platform to use transcriptomes for modern annotation. These tools allow to visualize gene expression profiles, draw molecular evolutionary phylogenetic trees of gene families, perform functional enrichment analyses, and facilitate output downloads. To demonstrate their utility, we analyzed the gene expression profiles of enzymes within the nicotine biosynthesis pathway, a secondary metabolic pathway characteristic of the *Nicotiana* genus. Using the developed tool, expression profiles of the nicotine biosynthesis pathway genes were generated. The expression patterns of eight gene groups in the pathway were strongly expressed in the roots and weakly expressed in leaves and flowers of *N. benthamiana*. The results were consistent with the established gene expression profiles in *Nicotiana tabacum* and provided insights into gene family composition and expression trends. The compilation of this database tool can facilitate genetic analysis of *N. benthamiana* in the future.

## Significance Statement

A tool was developed to visualize gene expression profiles, draw molecular evolutionary phylogenetic trees of gene families, perform functional enrichment analyses, and facilitate output downloads of *Nicotiana benthamiana*. The database developed using the tool can evolve into a comprehensive all‐in‐one analysis platform by continuously incorporating transcriptome data released to date, newly released RNA‐seq data, and annotations in the future.

## INTRODUCTION

*Nicotiana benthamiana* is one of the oldest experimental models used in plant biology (Bally et al., 2018; Bombarely et al., 2012) and is valuable in plant pathology studies, notably, because of its susceptibility to a wide range of plant diseases, especially viral diseases. *N. benthamiana* has also recently received attention as a general platform for recombinant protein production. For example, viral deconstructed vectors have proven highly efficient in producing recombinant proteins in *N. benthamiana* with a yield of approximately 4 mg g^−1^ fresh weight (Hoshikawa et al., 2019; Yamamoto et al., 2018). Furthermore, the production of a COVID‐19 vaccine has been achieved by inoculating and producing noninfectious virus‐like particles (Bally et al., 2018; Mamedov et al., 2021; van Herpen et al., 2010).

Interfamily grafting can also be realized in *N. benthamiana* (Kurotani & Notaguchi, 2021; Notaguchi et al., 2020). Although grafting is an old agricultural technique, its applicability has been limited, and it is considered to be established only among very closely related species in the same species or family. As *N. benthamiana* can achieve cell–cell adhesion at the graft junction with plants of different families, it has attracted attention as a target for studying self‐recognition and wound repair mechanisms among plants.

*Nicotiana benthamiana* is a member of the family Solanaceae, which includes many agriculturally important crop species, such as potatoes, tomatoes, eggplants, petunia, and tobacco. *N. benthamiana* is native to Australia and almost 75 *Nicotiana* species are distributed in America and Australia. *N. benthamiana* in the *Suaveolentes* section forms *n* = 19 allopolyploid, demonstrating that the paternal progeny belongs to the *Sylvestres* section (Bally et al., 2018; Knapp et al., 2004), whereas the maternal lineage may be the ancestor of the *Noctiflorae* section; however, the details are not clear. *Nicotiana tabacum*, the most widely cultivated and common tobacco species, is found in the *Nicotiana* section and descends from the ancestral species of the *Sylvestres* and *Tomentosae* sections. *De novo* whole‐genome assembly was performed in *N. benthamiana* using HiFi reads, creating 1668 contigs, 3.1 Gb in length. The 21 longest scaffolds are considered pseudomolecules containing 2.8 Gb of sequence. In total, 57 583 high‐confidence gene sequences were predicted within a 61 Mb region (Kurotani et al., 2023). Other studies achieved the construction of 19 pseudomolecules (Ko et al., 2024; Ranawaka et al., 2023). Transcription‐start‐site and transcription‐termination‐site sequencing provided accurate gene annotation (Wang et al., 2024). These results are complementary to each other.

In this study, we aimed to create a usable database of the gene expression profiles of *N. benthamiana* based on stored genomic information. In addition to the previously conducted time‐series transcriptomes of grafted plants, expression maps sampled by plant sites and site‐specific transcriptomes of wound‐treated or grafted plants were obtained to construct the database. By providing graphical expression profiles and a comprehensive set of analytical tools, including a sequence homology search, phylogenetic analysis, and heat mapping, it is expected that *N. benthamiana* will be more easily used as a model for plant science.

## RESULTS AND DISCUSSION

### Reconstruction of the *N. benthamiana* genome scaffold

Although our sequence analysis yielded 21 sufficiently long scaffolds, the genome of *N. benthamiana* is known to contain 19 pairs of chromosomes. Since other groups have later published genome analyses and showed 19 chromosome pairs (Ko et al., 2024; Ranawaka et al., 2023; Wang et al., 2024), we used the analysis in Ranawaka et al., 2023 as a reference to reconstruct our scaffold. We compared the sequences and found that 17 of our 21 pairs of scaffolds were nearly identical to the ones of Ranawaka et al., 2023. For the remaining four, two pairs could be linked as one set of chromosomes each. As a result, we were able to put together our scaffolds as 19 pairs of chromosomes (Figure S1). The orientation of each chromosome was changed to match to ones of Ranawaka et al. (2023) and the database was reconstructed. Genes were annotated in the order in which they were originally created, except for two pairs of chromosomes that were spliced together. Chromosome IDs of the linked chromosomes were reassigned as serial numbers (Figure S1b).

### Construction of *N. benthamiana* gene expression database

**Figure 1:**
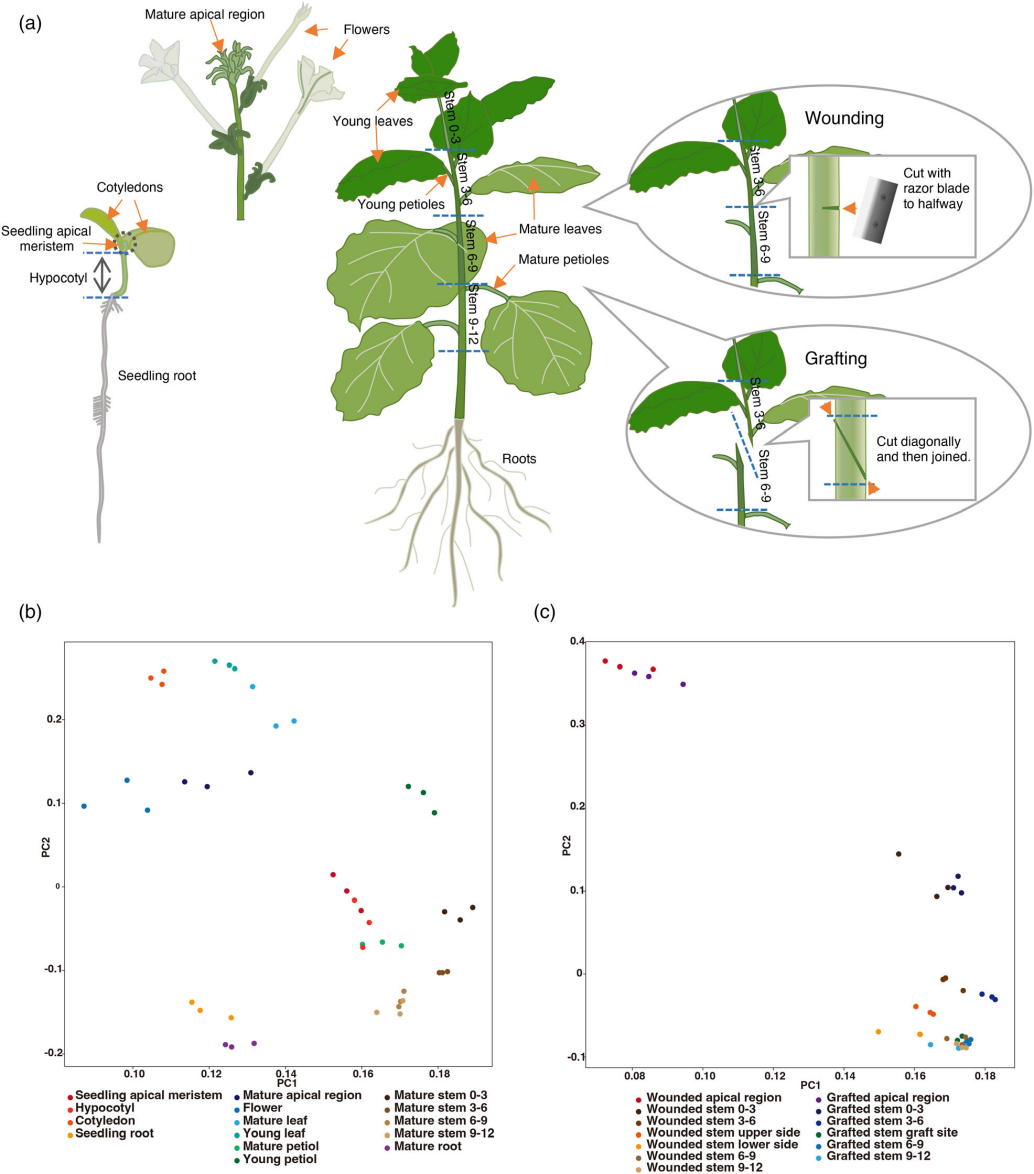
Transcriptome analysis by RNA‐seq. (a) Plants and their parts used for RNA‐seq analysis shown in the schematic illustration. (b, c) Principal component analysis for transcriptome. (b) Mature plants (7 weeks after germination) and seedling plants (7 days after germination). (c) Wounding or grafting (7 days after treatment).

To construct the *N. benthamiana* expression database, in addition to our previously published 11‐point time‐series transcriptome data of grafted plants, we performed transcriptome analysis of 28 new plant sites, yielding 117 RNA‐seq data sets (Figure 1a; Figure S2) of the stem apex, cotyledons, hypocotyls, and roots at the seedling stage; young and fully expanded leaf apical regions; floral organs; stems; and roots at the mature plant stage. These parts are selected as the key tissues and organs in the *N. benthamiana* life cycle. Site‐specific sectioned tissue for stems when grafted or wound‐treated to the stem, and RNA‐seq data over time for previously examined grafted plants were also included (Notaguchi et al., 2020). All RNA‐seq data were processed using the same pipeline to obtain transcripts per million (TPM) and raw count matrices. Principal component analysis (PCA) was performed for each of the three groups: intact plant samples at all growth stages, wound‐treated, and grafted 7‐day stems (Figure 1b,c). The transcriptomes of each of the three replicates were plotted at approximate positions, suggesting that the analyses were performed correctly. The leaves, roots, stems, petioles, and floral organs each formed four populations, reflecting the direction of differentiation (Figure 1b). The shoot apical meristems of seedlings were possibly not strictly separated from the hypocotyl. Stem samples on Day 7 of wound treatment and grafting were mostly similar and were plotted in similar positions for each stem site (Figure 1c). The wound and grafting treatments may have been nearly identical in terms of the physiological responses. PCA result of the time‐series transcriptome of the grafting sites showed a behavior consistent with the previous mapping to the Niben1.01 genomic reference; however, the movement of the plots along the time‐series depicted a more continuous linearity (Figure S3; Notaguchi et al., 2020). This may have been due to the annotation accuracy.

**Figure 2:**
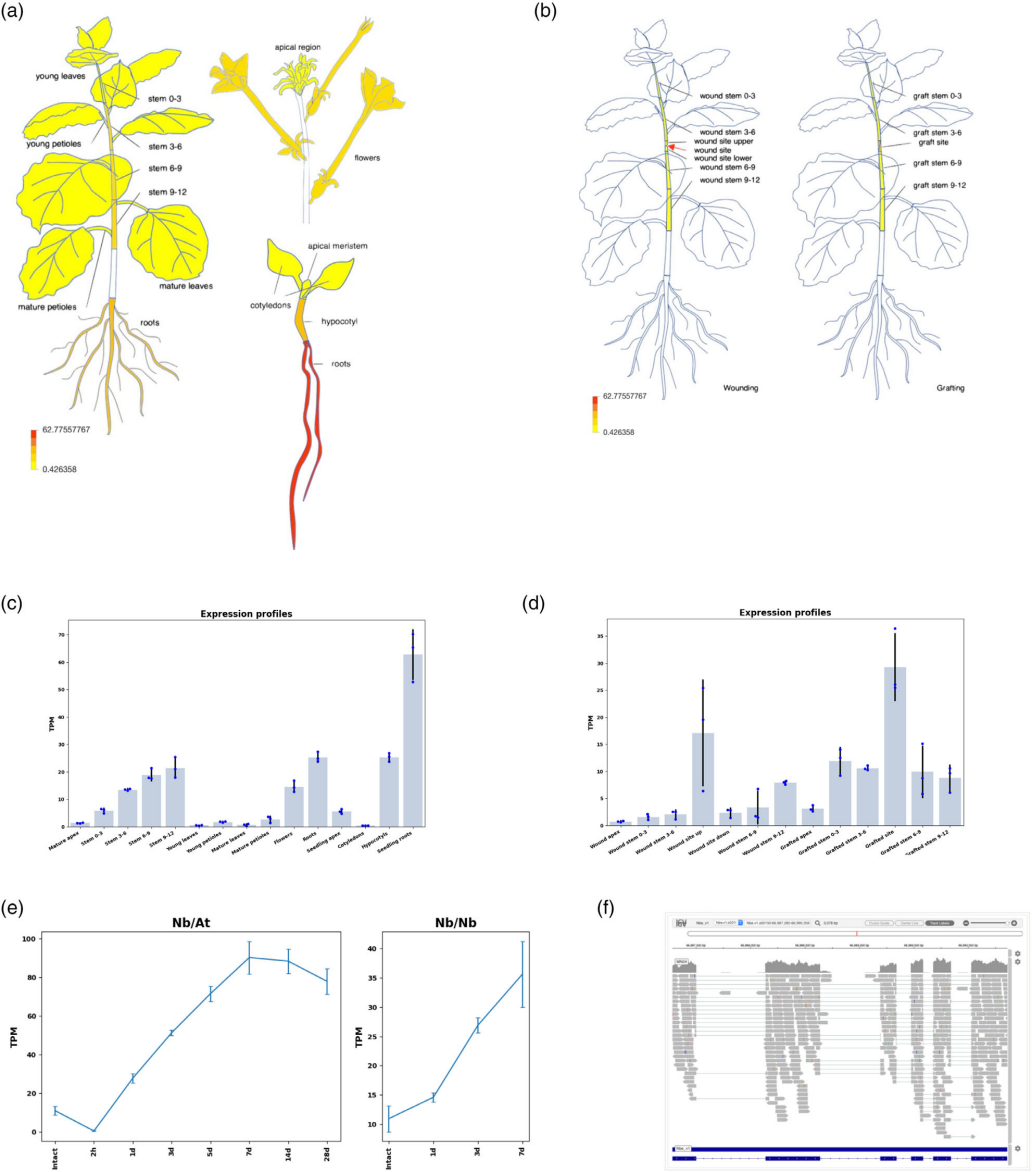
Construction of graphical expression browser. Examples of expression levels of *Nbe.v1.1.chr03g18250.1* gene. (a, b) Heat‐pictograph of gene expression levels (transcripts per million, TPM) by plant site in *Nicotiana benthamiana*. (a) Plants at 7 weeks after germination and seedling stages were separated by site. (b) Plants at 6 weeks after germination were wound‐treated (left) or grafted, and the expression was examined in the stems 7 days after treatment. (c, d) Expression levels for three replicates are shown in the bar graph with error bars. (c) and (d) are bar graphs of (a) and (b), respectively. (e) Time‐series transcriptome of grafted plants is displayed as a line graph. (f) The mapping status of the sequence reads from the RNA‐seq analysis used to calculate the expression level is displayed in the Genome Browser. Error bars indicate standard deviation.

The database provides tools for visualizing gene expression profiles, sequence homology searches, molecular phylogenetic tree generation from the obtained sequences, heat mapping, and hierarchical clustering of expression profiles (Figure S4). Each analysis tool allows the annotation list to be used as a hub for link analyses. Gene annotations can be searched using Nbe.v1 ID (Kurotani et al., 2023), Niben1.01 ID (Bombarely et al., 2012), and TAIR AT ID (Huala et al., 2001). A free‐word search using gene description in the Arabidopsis annotation is also supported. By directly specifying a list of annotations or annotation IDs, the expression profile of each gene can be displayed as a color heat‐pictograph (Figure 2). Figure 2 displays the expression profile of *GH9B3*, *beta 1,4‐glucanase* (*Nbe.v1.1.chr03g18250.1*) as an example. The expression profiles that can be displayed are divided into cotyledons, hypocotyls, roots, stems, petioles, leaves, flowers, and stem tips from budding, vegetative, and trophic individuals (Figure 2a,c). Expression profiles for each grafted or wounded stem site are also shown (Figure 2b,d). Simultaneously, users can display those profiles as bar graphs. The heat‐pictograph can be displayed as either the absolute value of expression (TPM) or a normalized *z*‐score, with the mean set to 0 and the standard deviation set to 1. The time‐series transcriptomes of grafted stems are only available as a graph (Figure 2e). Integrative Genomics Viewer (IGV, Robinson et al., 2011) was embedded to display the map status of sequence reads for all transcriptome data (Figure 2f). These expression profiles were constructed using the LAB strain of *N. benthamiana*, but the transcriptome data from an independent LAB strain that has previously been published and transcriptome data from another different strain, the QLD strain, were also made available for browsing (Figure S5; Ranawaka et al., 2023). To account for possible differences in mapping efficiency, a more relative comparison can be made with a *z*‐scored display.

### Construction of *N. benthamiana* gene search and sequence comparison tools

Blast+ of the National Center for Biotechnology Information (Camacho et al., 2009) was incorporated as a homology search tool for gene sequences. The coding sequence (CDS) is obtained from the annotation of the gene to be examined, and the search can be directly applied to the BLAST program. The search targets are Nbe.v1 and Niben1.01 for *N. benthamiana* and Araport11 of TAIR for *Arabidopsis thaliana*. Entire genome and transcript databases are available (https://nbenthamiana.jp). We implemented two search methods: Blastn, which searched the DNA database using the DNA sequence as a query, and tBlastn, which translated the query and DNA database into three frames of amino acids and searched only for transcripts. DNA sequences are entered directly as queries or FASTA format files are uploaded for analysis. The genes to be compared, obtained by BLAST, can be aligned with ClustalW using annotation IDs or their FASTA‐formatted sequences, and a molecular phylogenetic tree can be drawn using the neighbor‐joining method. The drawn images are provided in EPS format for easy processing and downloading (Figures S6–S8).

### Construction of *N. benthamiana* gene expression analysis tools

**Figure 3:**
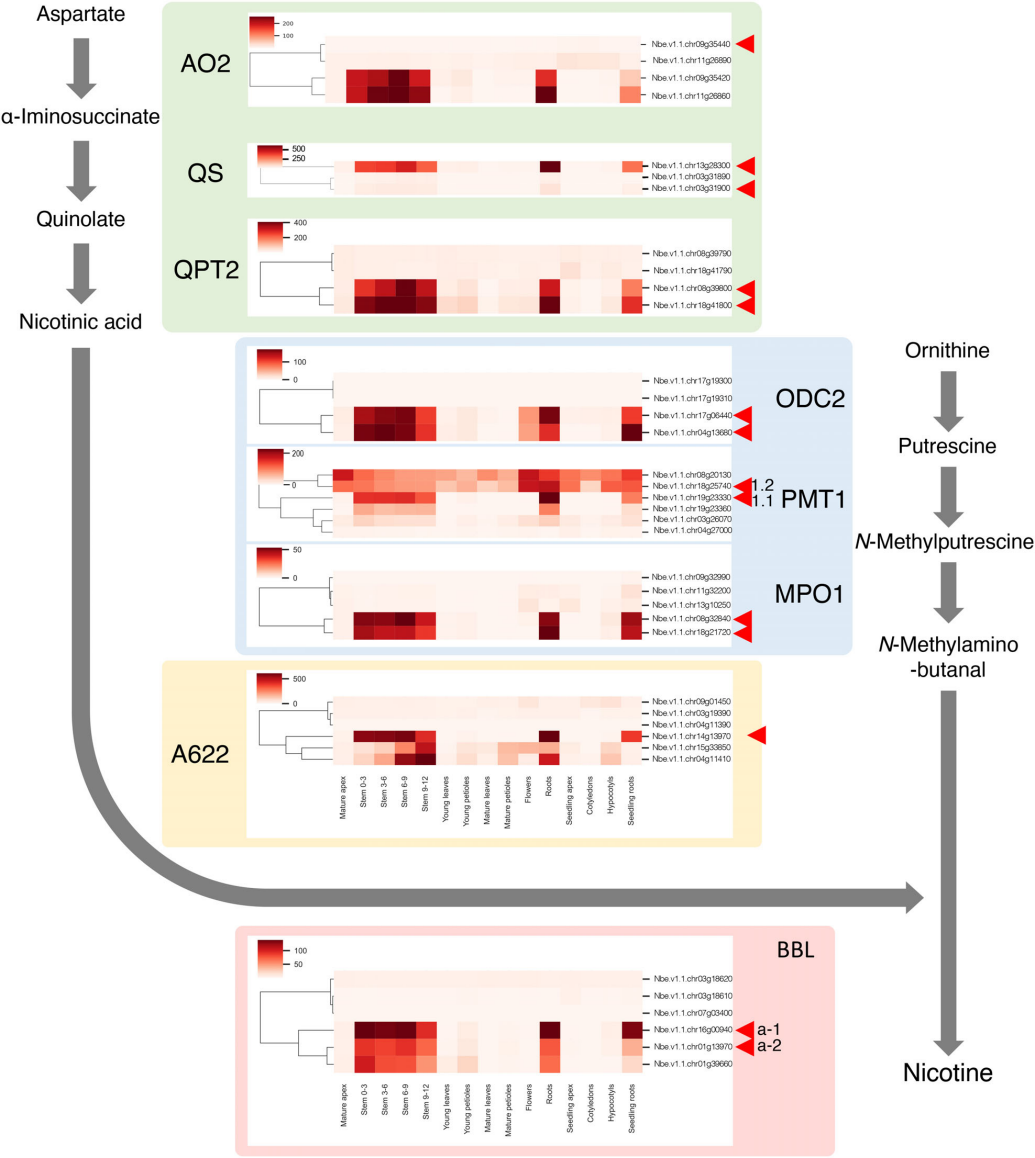
Expression analysis of a group of genes in the nicotine biosynthesis pathway. Profiles of *Nicotiana benthamiana* genes homologous to the eight genes involved in nicotine biosynthesis in *N. tabacum* were generated. Each gene is represented by a heat map, and a phylogenetic tree based on the expression profiles with Ward and Euclidian distance is placed to the left of the heat map. Genes that most closely approximated the *N. tabacum* genes are indicated by arrowheads.

A tool was prepared to create heat maps from the expression profiles of multiple genes, including gene families (Figure 3). This tool allows hierarchical clustering of genes from profiles. Clustering among samples was also made possible. The clustering algorithm can be selected from the Ward's, single linkage, complete linkage, average linkage, weighted, centroid, and median methods. Distance can be selected from Euclidean, correlation, cosine, and city blocks. *N. benthamiana*, an allopolyploid species, has many genes in pairs that are located in the genome of two interbred species (Bally et al., 2018); therefore, similar genes often form large families. Because of this tool, these genes can be easily organized and evaluated, thereby facilitating genetic analyses, including comprehensive genome editing, using CRISPR.

### Case study: expression profile analysis of nicotine biosynthesis pathway genes

To test the plausibility and utility of the developed database, we focused on the genes associated with nicotine biosynthesis in *N. benthamiana*. Nicotine is a major alkaloid and typological feature of plants of the genus *Nicotiana* and plays an important role in plant defense mechanisms (Hashimoto & Yamada, 1994; Steppuhn et al., 2004). Nicotine biosynthesis involves the pyridine pathway metabolized from aspartate and the pyrrolidine pathway metabolized from ornithine. Aspartate oxidase (AO), quinolinic acid synthase (QS), quinolinic acid phosphoribosyltransferase (QPT), and other enzymes are involved in the early stages of nicotinic acid biosynthesis (Katoh et al., 2006; Sinclair et al., 2000) and nicotinic acid‐derived precursors. Ornithine decarboxylase (ODC), putrescine *N*‐methyltransferase (PMT), and *N*‐methylptorescine oxidase (MPO) are involved in the formation of pyrrolidine rings (Heim et al., 2007; Hibi et al., 1994; Imanishi et al., 1998; Katoh et al., 2007). The phosphatidylinositol phosphate (PIP) family oxidoreductase A622 and the berberine bridge enzyme‐like protein (BBL) are suggested to be involved in the late bio‐synthetic steps of pyridine alkaloids (Deboer et al., 2009; Kajikawa et al., 2009; Lewis et al., 2015; Vollheyde et al., 2023). Phylogenetic analysis suggests that the genes involved in the biosynthesis of pyridine and pyrrolidine rings evolved from the duplication of two major metabolic pathways that have long existed in all major plant lineages: the nicotinamide adenine nucleotide (NAD) coenzyme pathway and the polyamine metabolism pathway, respectively (Xu et al., 2017). Transposons, which are characteristics of the Solanaceae family, have been suggested to cause the evolutionary duplicating of these genes and their differentiation into pathways (NAD/pyridine ring pathways and polyamine/pyrrolidine ring pathways) with different regulations of expression and function (Xu et al., 2017).

In the present study, we first examined the genetic components of the nicotine biosynthetic pathway genes in *N. tabacum* and their homologs in *N. benthamiana* (Figures S6–S8). Homologs of *N. benthamiana* were searched by performing homology analysis in blast using the amino acid sequence of one molecular species of the relevant gene in *N. tabacum* as a query. Homologous genes were identified in the same way for the gene in *N. tabacum*, and by performing phylogenetic analyses of these genes together, homologs of *N. benthamiana* for each molecular species were determined. Both *N. benthamiana* and *N. tabacum* have allopolyploid genomes, with many genes having two copies derived from the original parent's genome. In addition, the homology of the two copies of *N. tabacum* is often closer than the homology of the homologs of *N. benthamiana* and *N. tabacum*, due to the fact that *N. benthamiana* is quite old when it became allopolyploid. Therefore, when phylogenetic trees are generated, *N. benthamiana* and *N. tabacum* form independent clades, and it is often difficult to strictly identify the homologs. In this study, using the genome sequence and annotation of Sierro et al. (2024) as the gene sequence of *N. tabacum*, and if the same clade contains one gene each for *N. tabacum* and *N. benthamiana*, they are selected as homologs, and when a clade contains multiple genes, the pair with the shortest total length of tree branches is selected as homologs.

Two *AO* genes were in the pyridine ring pathway, *NtAO1* and *NtAO2*, in *N. tabacum*. *NtAO2* was reported to be involved in nicotine biosynthesis. The homologs of *N. benthamiana* contained four genes. *Nbe.v1.1.chr11g26890.1* is in the same clade as *NtAO2* and is the closest match, but there is another gene for *N. tabacum* in the same clade; neither *N. tabacum* nor *N. benthamiana* genes are in clear pairs in the phylogenetic tree. It is difficult to determine which is the homolog of each (Figure S6a).

*QS* is suggested to share a common gene that functions in the NAD and pyridine ring pathways. Two copies of this gene are present in *N. tabacum*. Three genes were found in *N. benthamiana* that could be homologs of them: the homolog of *Ntab04g010910‐1.1* was considered to be *Nbe.v1.1.chr03g31900.1* and the one most homologous to *Ntab06g021890‐1.1* was *Nbe.v1. 1.chr13g28300.1* (Figure S6b).

There were two *QPT* genes, *NtQPT1* and *NtQPT2*, in *N. tabacum*, and the genes most closely related to *NtQPT1* and *NtQPT2* were *Nbe.v1.1.chr08g39790.1* and *Nbe.v1.1.chr08g39800.1* in *N. benthamiana*, respectively (Figure S6c). However, QPT2 is considered to have two genes in *N. benthamiana*, and *Nbe.v1.1.chr08g39800.1* is called *NbQPT2a* and *Nbe.v1.1.chr18g41800* is called *NbQPT2b*.

In the pyrrolidine ring pathway, *Nbe.v1.1.chr04g13680.1* and *Nbe.v1.1.chr17g06440.1* were found as genes homologous to *NtODC1* and *NtODC2* of *N. tabacum*, respectively (Figure S7a). Since *Nbe.v1.1.chr04g13680.1* also belongs to the same clade as *NtODC2*, *Nbe.v1.1.chr17g06440.1* is designated as *NbODC2a* and *Nbe.v1.1.chr04g13680.1* as *NbODC2b*.

In *N. tabacum*, *NtPMT1.1* and *NtPMT1.2* have been reported to function in the nicotine biosynthesis pathway as *PMT1* genes (Xu et al., 2017). In this study, we investigated the *PMT1.1* and *PMT1.2* genes in *N. tabacum* and found that *NtPMT1.1* in particular has four genes with nearly identical CDS sequences. Similarly, five genes with nearly identical sequences were found in *N. benthamiana*, but it was difficult to map them to the four genes in *N. tabacum*. The closest homolog to *N. tabacum PMT1.1s* was *Nbe.v1.1.chr19g23330.1*, so it is referred to as *NbPMT1.1* in this study. The homolog of *NtPMT1.2* was *Nbe.v1.1.chr18g25740.1* (Figure S7b).

*MPO* was found in *N. tabacum* with five genes, and in *N. benthamiana* with five genes. From the phylogenetic tree, several *NtMPOs* could be identified with corresponding *N. benthamiana* homologs, but no clear clade was formed for *NtMPO1*. Based on branch length, we designated two genes, *Nbe.v1.1.chr18g21720.1* and *Nbe.v1.1.chr08g32840.1*, as *NbMPO1a* and *NBPMO1b*, respectively. (Figure S7c).

A622, the enzyme involved in the reaction that joins the two rings, has two genes, *NtA622* and *NtA622‐like*, in *N. tabacum*. Although many genes similar to these are found in *N. benthamiana*, we selected *Nbe.v1.1.chr14g13970.1* as the gene that belongs to the same clade in the phylogenetic tree and is most similar to *NtA622* (Figure S8a).

In *N. tabacum*, four *BBL* genes (*NtBBLa*–*NtBBLd*) were reported to be involved in this pathway. In the latest *N. tabacum* genome, these were annotated with six genes, and the same six genes were found in *N. bentamiana*. However, it was difficult to map these six genes strictly based on phylogenetic tree analysis. *Nbe.v1.1.chr01g13970.1* and *Nbe.v1.1.chr16g00940.1* were designated *NbBBLa‐1* and *NbBBLa‐2* as homologs of *NtBBLa* and *Nbe.v1.1.chr07g03400.1* was designated as *NbBBLd* (Figure S8b).

The expression profiles of these nicotine biosynthesis pathway genes were generated using the tool developed in this study (Figure 3). The expression patterns of all eight gene groups were strongly expressed in the roots and weakly expressed in the leaves and flowers, as previously reported for *N. tabacum* (Xu et al., 2017). The hypothesis of the molecular evolution of nicotine biosynthetic pathways through gene duplicating during the evolution to the *Nicotiana* genus in the Solanaceae family was also generally supported. The only difference was in the AOs, which are the primary enzymes in the pyridine ring pathway. In *N. tabacum*, NtAO2 metabolizes aspartate to α‐iminosuccinate (Xu et al., 2017). However, the genes are not as well expressed as those more homologous to *NtAO2*, suggesting that the functional differentiation of these genes may have been acquired independently after establishing the two species.

A comparison was performed for two genes, *BBLa‐1* and *BBLa‐2*, using RNA‐seq data of Australian LAB and QLD strains that had previously been collected. The expression profiles of the LAB strain obtained in this study and those of the Australian LAB strain showed similar expression patterns. In addition, the expression profiles of these genes were found to be comparable to those of the QLD strain (Figure S9).

### Transcriptome analysis of *N. benthamiana* infiltrated with *Agrobacterium tumefaciens*

**Figure 4:**
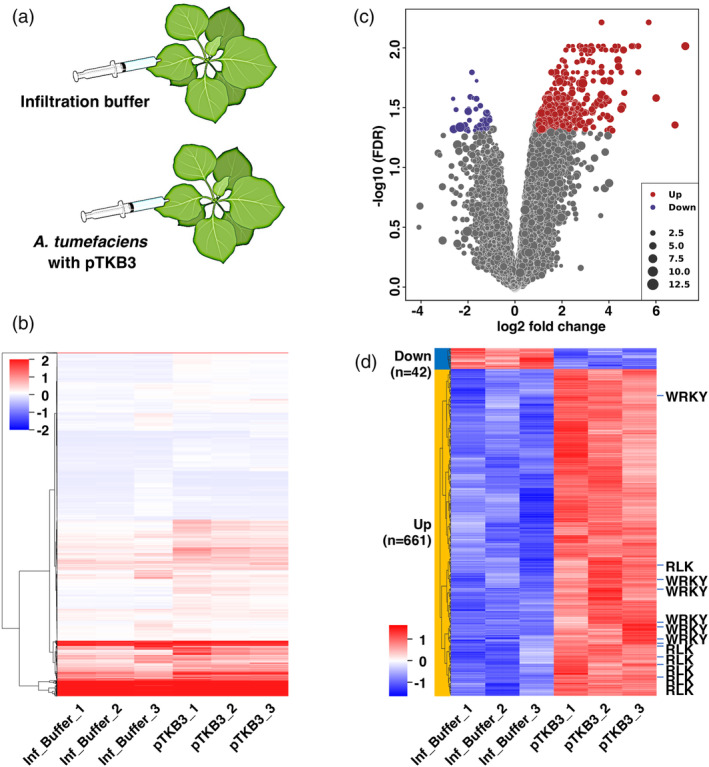
Transcriptome analysis of *Nicotinana benthamiana* agroinfiltration. Transcriptomes of *N. benthamiana* genes following infiltration with *Agrobacterium tumefaciens* GV3101 containing the binary vector pTKB3 and infection buffer control. (a) Schematic image of agroinfiltration in *N. benthamiana*. (b) A hierarchical clustering heat map of the gene expression. Clustering was performed for the top 10% of genes with the largest standard deviation of expression levels (TPM) per gene between samples. The gene expression data was clustered using *z*‐scores with Ward and Euclidean distance. (c, d) Differential gene expression analysis between buffer control and agroinfected leaves was performed using limma package with an FDR cutoff of 0.05 and a minimum fold change of 2 (Love et al., 2014). (c) Volcano plot of gene expression profile data. The red, blue, and gray dots represent upregulated genes, downregulated genes, and nondifferentially expressed genes (DEG), respectively. The size of dots indicates the average expression level of each gene. (d) DEG heatmap. Genes shown on the right indicate WRKY transcription factors and receptor‐like kinases (RLK), which were detected particularly frequently. Inf_Buffer and pTKB3 indicate infiltration of buffer solution and GV3101 with pTKB3 vector, respectively.

We also conducted a comprehensive RNA‐seq analysis to investigate the transcriptomic changes in *N. benthamiana* following infiltration with *A. tumefaciens* harboring the pTKB3 vector, compared to plants infiltrated with the infiltration buffer (Figure 4a,b). Differential expression gene (DEG) analysis revealed that agroinfiltration significantly alters gene expression and identified 641 genes was significantly upregulated (*P* < 0.05), and 42 genes were significantly downregulated (*P* < 0.05) (Figure 4c,d). The upregulated genes included a diverse array of functions, as expected genes related to protein synthesis machinery including several ribosomal proteins and amino acids‐tRNA conjugation enzymes, regulation of DNA replication and repair, and regulation of programmed cell death. Interestingly, genes involved in protein processing and degradation like ubiquitin ligases, as well as those associated with defense responses like receptor‐like kinases and WRKY transcription factors, were markedly upregulated (Figure 4d; Figures S10–S12; Table S2). To enhance recombinant protein yields beyond current limitations, one promising strategy involves manipulating *A. tumefaciens* pathogen‐associated molecular patterns (PAMPs) or the plant's PAMP receptors to reduce the immune response, thereby improving the efficiency and yield of recombinant protein production (Saur et al., 2016; Yang et al., 2023). Therefore, the findings highlighted here are essential not only for gaining insights into plant–pathogen interactions but also for optimizing recombinant protein production, thereby advancing plant biotechnology.

### Conclusion and future prospects

The *N. benthamiana* database can perform a series of analyses, from annotation and sequence homology searches to web‐based data visualization. This database is expected to evolve into a comprehensive all‐in‐one analysis platform by continuously incorporating transcriptome data released to date, newly released RNA‐seq data, and annotations in the future. Currently, tools for differentially expressed gene analysis from expression data, expression network analysis, and pathway analysis have not yet been implemented. This database is expected to accelerate the molecular biological discovery of *N. benthamiana* and make *N. benthamiana* an easier‐to‐use plant research platform than ever before.

## EXPERIMENTAL PROCEDURES

### Plant materials

*Nicotiana benthamiana* seeds were surface‐sterilized with 5% (w/v) bleach for 5 min, washed three times with sterile water, incubated at 4°C for 3 days, and sown on half‐strength Murashige and Skoog medium supplemented with 0.5% (m/v) sucrose and 1% agar. The pH was adjusted to pH 5.8 using 1 m KOH. *N. benthamiana* seedlings were grown at 27°C under continuous illumination intensity of 100 μmol m^−2^ sec^−1^. On Day 7 after germination, the plants were replanted in a 1:1 mixture of soil medium and vermiculite. Stem grafting was performed as previously described (Notaguchi et al., 2020). Briefly, wedge grafting was performed on stems, and the grafted plants were initially grown in an incubator at 27°C under continuous light (ca. 30 μmol m^−2^ sec^−1^) for a week and then transferred to a plant growth room at 22°C under continuous light conditions (ca. 80 μmol m^−2^ sec^−1^). Stem wounding was performed under the same conditions as grafting without complete disconnection. For *A. tumefaciens* infiltration assays, *N. benthamiana* seeds were planted on rockwool. Seedlings were grown under a photoperiodic condition (16‐h light/8‐h dark cycle of white light 100 μmol m^−2^ sec^−1^) at 23°C in a plant growth chamber. Six‐week‐old plants were used for infiltration at the third leaves counting top‐down starting with the youngest mature leaf.

### *Agrobacterium* infiltration and RNA extraction

Preparation of *A. tumefaciens* suspension and infiltration in *N. benthamiana* was performed as previously described (Yamamoto et al., 2018) with modifications. *A. tumefaciens* GV3101 containing the binary vector pTKB3 was grown in L‐broth media with antibiotics (100 mg L^−1^ of kanamycin, 30 mg L^−1^ gentamycin, and 30 mg L^−1^ of rifampin) for 2 days at 28°C. Then, 2‐day cultures were diluted 100 times in the same media with antibiotics, 10 mm MES (pH 5.6), and 20 μm acetosyringone, and grown for 18–24 h at 28°C on a rotary shaker at 140 rpm. After centrifugation at 4,000 x g for 10 min at 4°C, *A. tumefaciens* was resuspended in the infiltration buffer (10 mm MgCl_2_, 10 mm MES [pH 5.6], 100 μm acetosyringone) to adjust OD_600_ = approximately 1 and used for infiltrating three leaves of three different plants using a syringe. Leaves infiltrated with infiltration buffer only were used as a control. After infiltration, *N. benthamiana* plants were incubated at 23°C under a 16‐h light/8‐h dark photoperiod for 1 day, and then total RNA was extracted from 100 mg leaf samples using TRIzol reagent (Thermo Fisher Scientific, Waltham, MA, USA), according to the manufacturer's instructions.

### Transcriptomic analysis

Plants growing to approximately 15 cm in length 6–7 weeks after sowing were considered mature plants. Samples were taken from fully expanded mature leaves and their petioles, immature leaves and their petioles during elongation, stems cut from the stem apex every 3 cm, roots, flowering organs, and immature inflorescence meristems. Three replicates of 10 plant samples per pool were used for the analysis. Cotyledons, shoot apical meristems, hypocotyls, and roots were sampled from the seedlings 7 days after sowing. Three replicate analyses were performed using one tissue pool from 20, 100, 100, and 25 individuals, respectively. Wound treatments and grafting were performed on the plants 4–5 weeks after sowing. For wound treatment, a razor blade was used to cut 6 cm from the stem apex to half the stem diameter, and sampling was performed after 7 days. The top and bottom of the wounds were located 1 cm above and below each cut, respectively, and the remaining stems were removed every 3 cm. Similarly, during grafting, stems were cut diagonally 6 cm from the stem apex, joined using grafting clips, and sampled on Day 7. The grafted portion was sampled at a length of 1 cm without separating the upper and lower portions, and the remaining stems were cut into 3‐cm pieces. Three replicates of 10 plants per pool were used for the analysis. Tissues were processed with zircon beads and lysate binding buffer containing sodium dodecyl sulfate instead of lithium dodecyl sulfate, as described in a previous report. RNA purification and cDNA library preparation followed the BrAD‐seq method (Townsley et al., 2015). Further, 86‐bp single‐ended sequencing was performed on an Illumina NextSeq 500 platform (Illumina, San Diego, CA, USA). Data pre‐processing was performed as follows. Data were trimmed for quality using Fastp v0.23.2 (Chen et al., 2018) with the settings *q*‐score of 20 and length of 20. Trimmed reads were mapped onto the genome assembly using HISAT2 v2.1.0 (Kim et al., 2019). The generated sequence alignment and map (SAM) files were converted to binary alignment and map (BAM) format and merged using SAMtools v1.4.1 (Danecek et al., 2021). Gene expression levels (TPM) were estimated using StringTie v2.2.0 (Pertea et al., 2015).

### Browser tools

The backend of the expression database was implemented in Python using the Flask Web framework. Data were stored in SQlite3 database. The front end was developed using a bootstrap framework. The Integrative Genomics Viewer (IGV) was used to visualize the RNA‐seq reads (Robinson et al., 2011). To normalize the expression levels, SciPy and scikit‐learn libraries were used in Python (Pedregosa et al., 2012; Virtanen et al., 2020).

### Expression analysis and sequence comparison tools

The scipy.cluster.hierarchy.linkage, matplotlib, and seaborn.clustermap libraries were used for heat mapping and hierarchical clustering of expression levels (Hunter, 2017; Virtanen et al., 2020; Waskom, 2021). BLAST+ was used to search for annotations or homologies in gene sequences (Camacho et al., 2009). Only DNA sequences were used for queries, and Blastn and tBlastx were implemented. The search targets were Nbe.v1.1 and Niben1.01 for *N. benthamiana* and Araport11 for Arabidopsis, and both CDS or transcripts, and genomes were prepared. ClustalW2 (Thompson et al., 1994), which creates molecular phylogenetic trees from the annotation search results of multiple genes or FASTA format files containing multiple gene sequences, was incorporated. Google Library was used for gene ontology enrichment analysis from the annotation list (Klopfenstein et al., 2018).

## AUTHOR CONTRIBUTIONS

K‐iK and MN conceived the research and designed the experiments. K‐iK, HH, JA, CW, PMW, and SI rearranged the *N. benthamiana* genome. K‐iK, HH, KS, KT, MM, YI, TS, YT, AR, and JA performed the sample preparation and analyzed data with the support of YN, SI, KM, CW, PMW, and MN. K‐iK, AR, and MN wrote the manuscript.

## CONFLICT OF INTEREST

The authors declare no conflicts of interest.

## Supporting information


**Figure S1.** Comparison between Nbe.v1 and NbLab360 genome sequence. (a) Nbe.v1 original data and NbLab360. (b) Rearranged Nbe.v1.1 and NbLab360.
**Figure S2.** Plants and their parts used for RNA‐seq analysis. Bars: 1 cm.
**Figure S3.** PCA analysis for the time series transcriptome of interfamily grafting and homo grafting. *Nb*/*At* and *Nb*/*Nb* indicate interfamily grafting and homo grafting, respectively. RNA was extracted from grafted plants at 2 h after grafting (HAG) and 1, 3, 5, 7, 10, 14 and 28 days after grafting (DAG).
**Figure S4.** Database construction diagram.
**Figure S5.** Graphical expression browser for LAB and QLD strain. RNA‐seq data analyzed using RNA extracted from LAB and QLD strains were mapped to Nbe.v1.1 genome sequence constructed in this study as a reference. Display of *Nbe.v1.1.chr03g18250.13* with *z*‐scoring.
**Figure S6.** Molecular phylogenetic trees of genes on the pyridine ring pathway. (a) Aspartate oxidase (AO). (b) Quinolinic acid synthase (QS). *Nbe.v1.1.chr03g31890.1* was excluded from the phylogenetic tree because it is extremely short compared to the other genes. (c) Quinolinic acid phosphoribosyltransferase (QPT).
**Figure S7.** Molecular phylogenetic trees of genes on the pyrrolidine ring pathway. (a) Ornithine decarboxylase (ODC). *Nbe.v1.1.chr17g19300.1* and *Nbe.v1.1.chr17g19310.1* were excluded from the phylogenetic tree because they are quite shorter than the other genes. (b) Putrescine *N*‐methyltransferase (PMT). (c) *N*‐methylptoresine oxidase (MPO).
**Figure S8.** Molecular phylogenetic trees of genes on the late bio‐synthetic steps of pyridine alkaloids. (a) Phosphatidylinositol phosphate (PIP) family oxidoreductase A622. (b) Berberine bridge enzyme‐like protein (BBL).
**Figure S9.**. Comparison of expression levels of *NbBBLa* genes in different stocks and different strains. All RNA‐seq data from the LAB strain used in the study in Japan (LAB_Jpn) and the LAB and QLD strains used in Australia (LAB_Aus and QLD_Aus, respectively) were mapped to Nbe.v1.1 as reference genome sequence. The expression of *NbBBLa‐1* and *NbBBLa‐2* were compared. “leaf” in LAB_Jpn was the mean of “mature leaf,” “mature petiole,” “young leaf,” “young petiole,” and “stem” was the mean of “stem 0–3,” “stem 3–6,” “stem 6–9,” and “stem 9–12.”
**Figure S10.** Gene Ontology enrichment analysis of biological processes for upregulated genes between buffer control and agroinfiltrated leaves.
**Figure S11.** Gene Ontology enrichment analysis of cellular components for upregulated genes between buffer control and agroinfiltrated leaves.
**Figure S12.** Gene Ontology enrichment analysis of molecular functions for upregulated genes between buffer control and agroinfiltrated leaves.

**Table S1.** Annotation list.
**Table S2.** DEG analysis between buffer control and agroinfiltrated leaves.

## Data Availability

The genome assembly data, annotations and gene models, and transcriptome data are available at the NbenBase (https://nbenthamiana.jp). The obtained transcriptome sequences are available in the DNA Data Bank of Japan (www.ddbj.nig.ac.jp), under the accession numbers DRA017203 and DRA019141. For LAB and QLD strain transcriptome, BioProject PRJNA881799 on NCBI Sequence Read Archive (SRA) was used.
