# Supplementary material for: Establishing a comprehensive web‐based analysis platform for Nicotiana benthamiana genome and transcriptome
Source: Plant J. 2024 Dec 3;121(1):e17178. doi: 10.1111/tpj.17178 (PMC11712010; doi:10.1111/tpj.17178)
Supplement: Supplementary file 1 — Figure S1. Comparison between Nbe.v1 and NbLab360 genome sequence. (a) Nbe.v1 original data and NbLab360. (b) Rearranged Nbe.v1.1 and NbLab360. Figure S2. Plants and their parts used for RNA‐seq analysis. Bars: 1 cm. Figure S3. PCA analysis for the time series transcriptome of interfamily grafting and homo grafting. Nb/At and Nb/Nb indicate interfamily grafting and homo grafting, respectively. RNA was extracted from grafted plants at 2 h after grafting (HAG) and 1, 3, 5, 7, 10, 14 and 28 days after grafting (DAG). Figure S4. Database construction diagram. Figure S5. Graphical expression browser for LAB and QLD strain. RNA‐seq data analyzed using RNA extracted from LAB and QLD strains were mapped to Nbe.v1.1 genome sequence constructed in this study as a reference. Display of Nbe.v1.1.chr03g18250.13 with z‐scoring. Figure S6. Molecular phylogenetic trees of genes on the pyridine ring pathway. (a) Aspartate oxidase (AO). (b) Quinolinic acid synthase (QS). Nbe.v1.1.chr03g31890.1 was excluded from the phylogenetic tree because it is extremely short compared to the other genes. (c) Quinolinic acid phosphoribosyltransferase (QPT). Figure S7. Molecular phylogenetic trees of genes on the pyrrolidine ring pathway. (a) Ornithine decarboxylase (ODC). Nbe.v1.1.chr17g19300.1 and Nbe.v1.1.chr17g19310.1 were excluded from the phylogenetic tree because they are quite shorter than the other genes. (b) Putrescine N‐methyltransferase (PMT). (c) N‐methylptoresine oxidase (MPO). Figure S8. Molecular phylogenetic trees of genes on the late bio‐synthetic steps of pyridine alkaloids. (a) Phosphatidylinositol phosphate (PIP) family oxidoreductase A622. (b) Berberine bridge enzyme‐like protein (BBL). Figure S9.. Comparison of expression levels of NbBBLa genes in different stocks and different strains. All RNA‐seq data from the LAB strain used in the study in Japan (LAB_Jpn) and the LAB and QLD strains used in Australia (LAB_Aus and QLD_Aus, respectively) were mapped to Nbe.v1.1 as reference [file TPJ-121-0-s001.pdf]

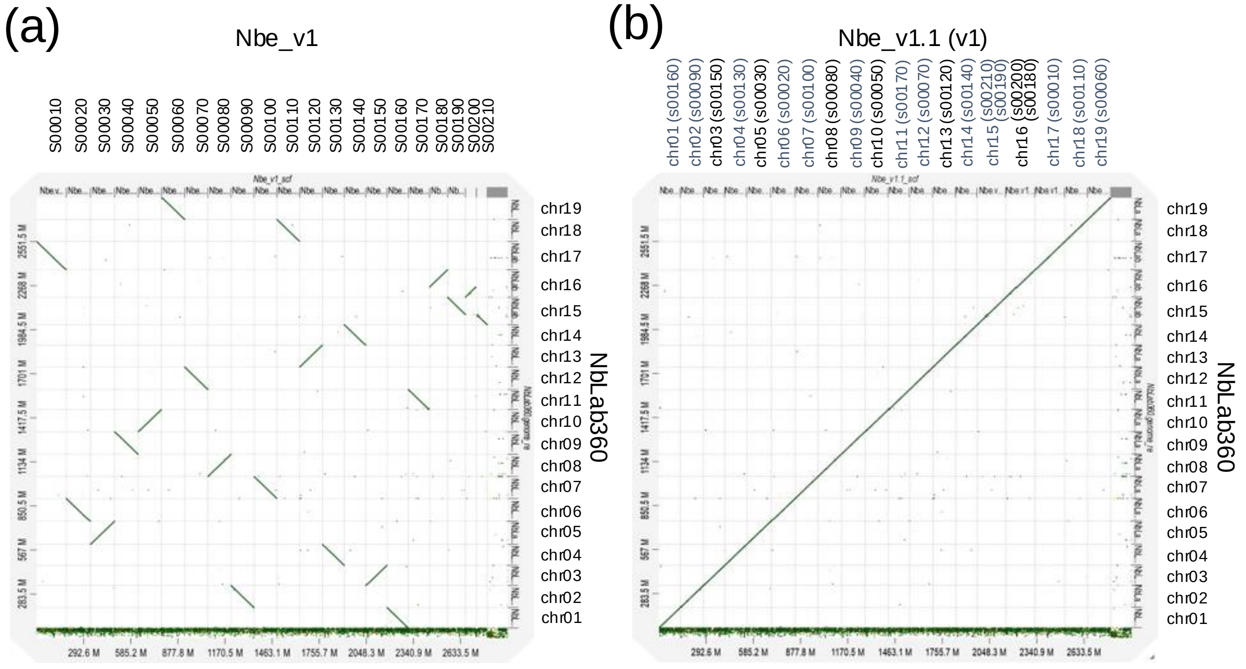

Supplementary Figure 1. Comparison between Nbe.v1 and NbLab360 genome sequence. (a) Nbe.v1 original data and NbLab360. (b) Rearranged Nbe.v1.1 and NbLab360.

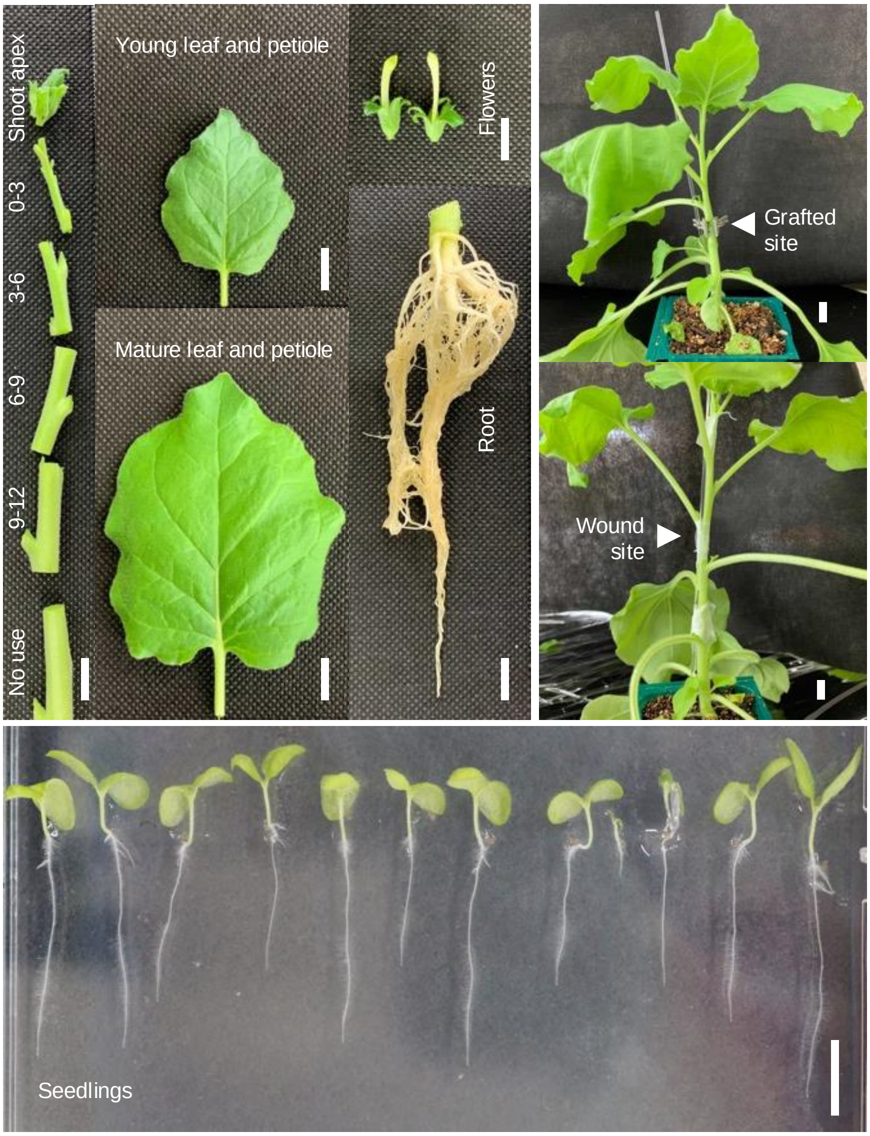

Supplementary Figure 2. Plants and their parts used for RNA-seq analysis
Bars:1 cm

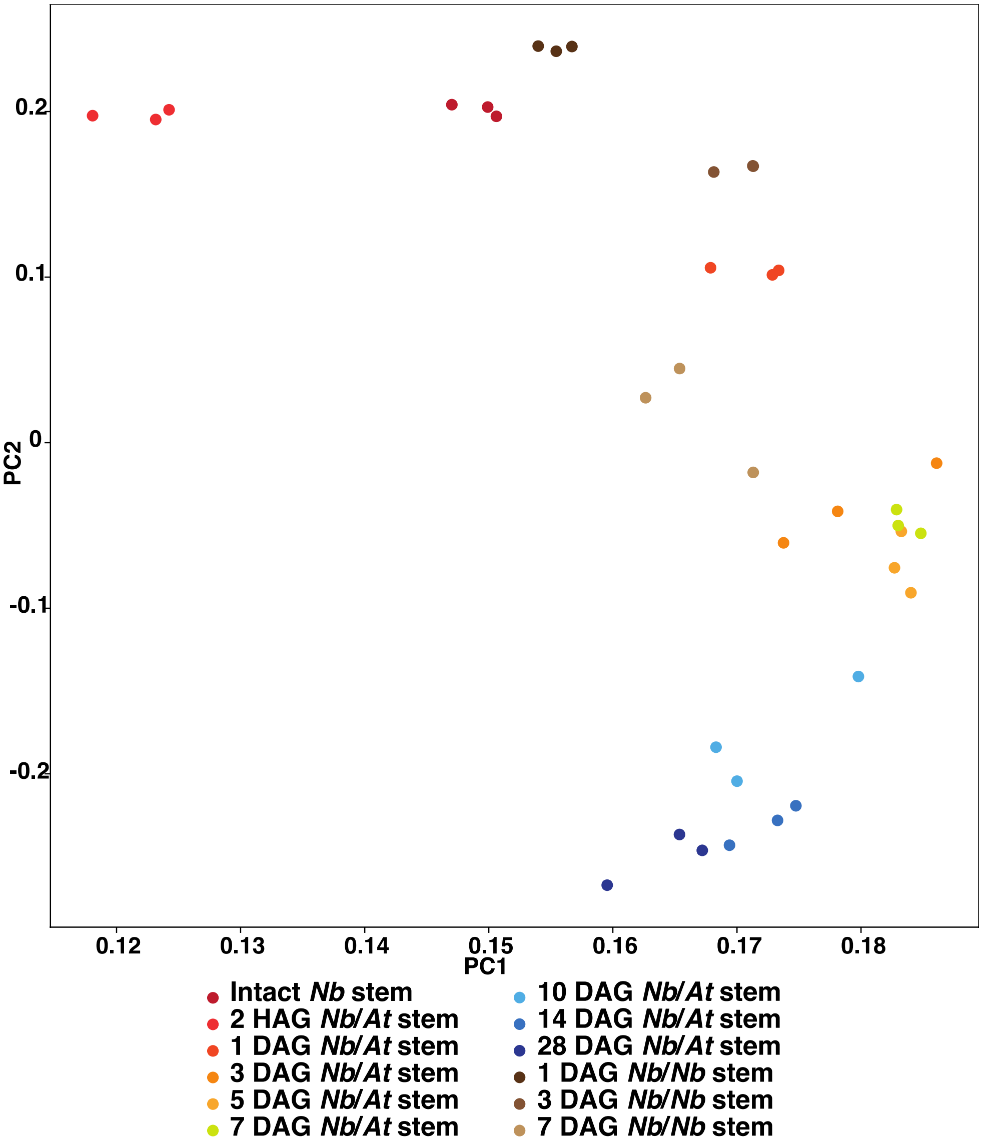

Supplementary Figure 3. PCA analysis for the time series transcriptome of interfamily grafting and homo grafting.

Nb/*At* and *Nb*/*Nb* indicate interfamily grafting and homo grafting, respectively. RNA was extracted from grafted plants at 2 hours after grafting (HAG) and 1, 3, 5, 7, 10, 14 and 28 days after grafting (DAG).

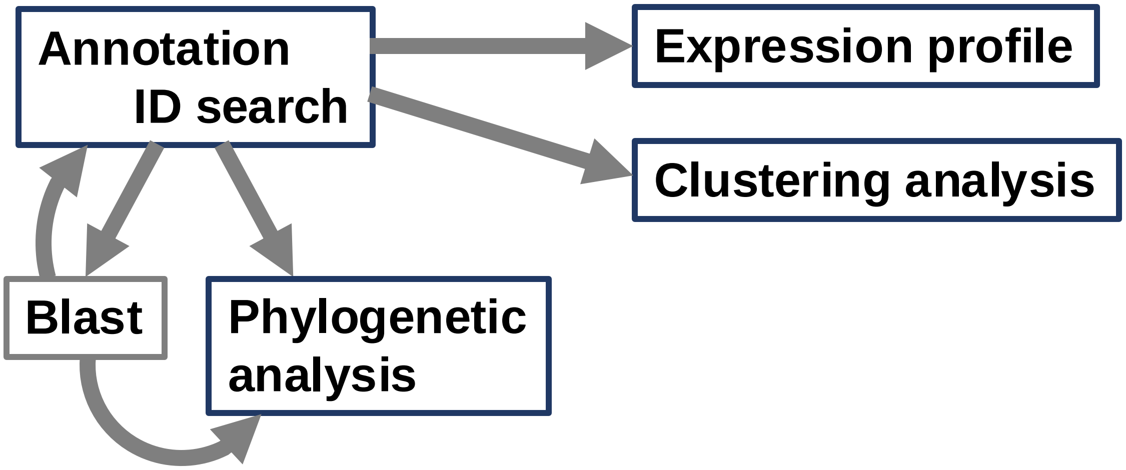

Supplementary Figure 4. Database construction diagram

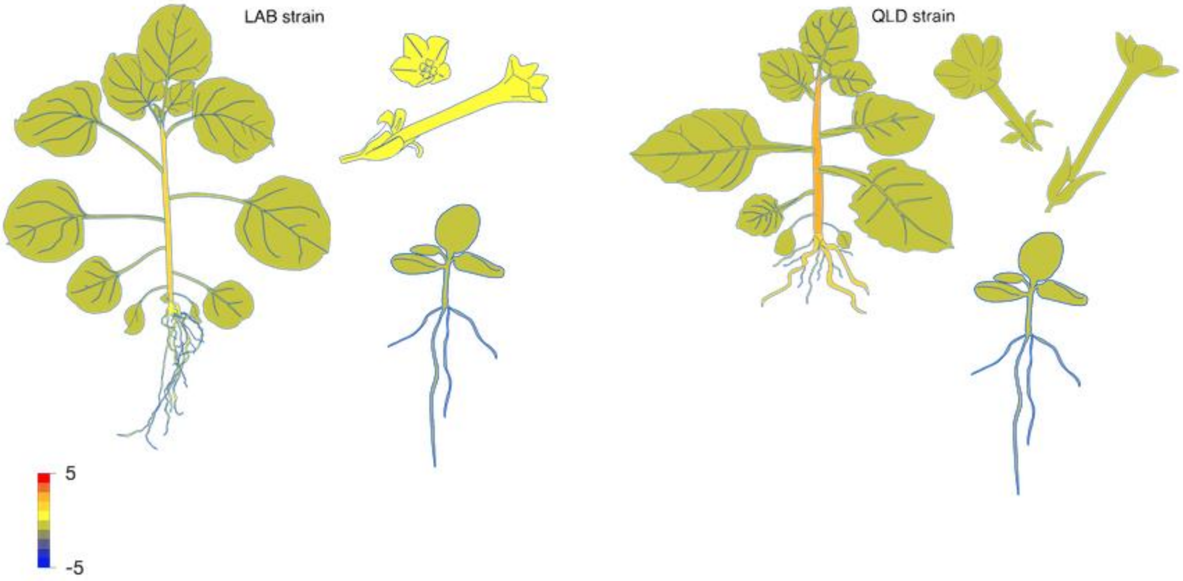

Supplementary Figure 5. Graphical expression browser for LAB and QLD strain
RNA-seq data analyzed using RNA extracted from LAB and QLD strains were mapped to Nbe.v1.1 genome sequence constructed in this study as a reference. Display of *Nbe.v1.1.chr03g18250.1* with z-scoring.

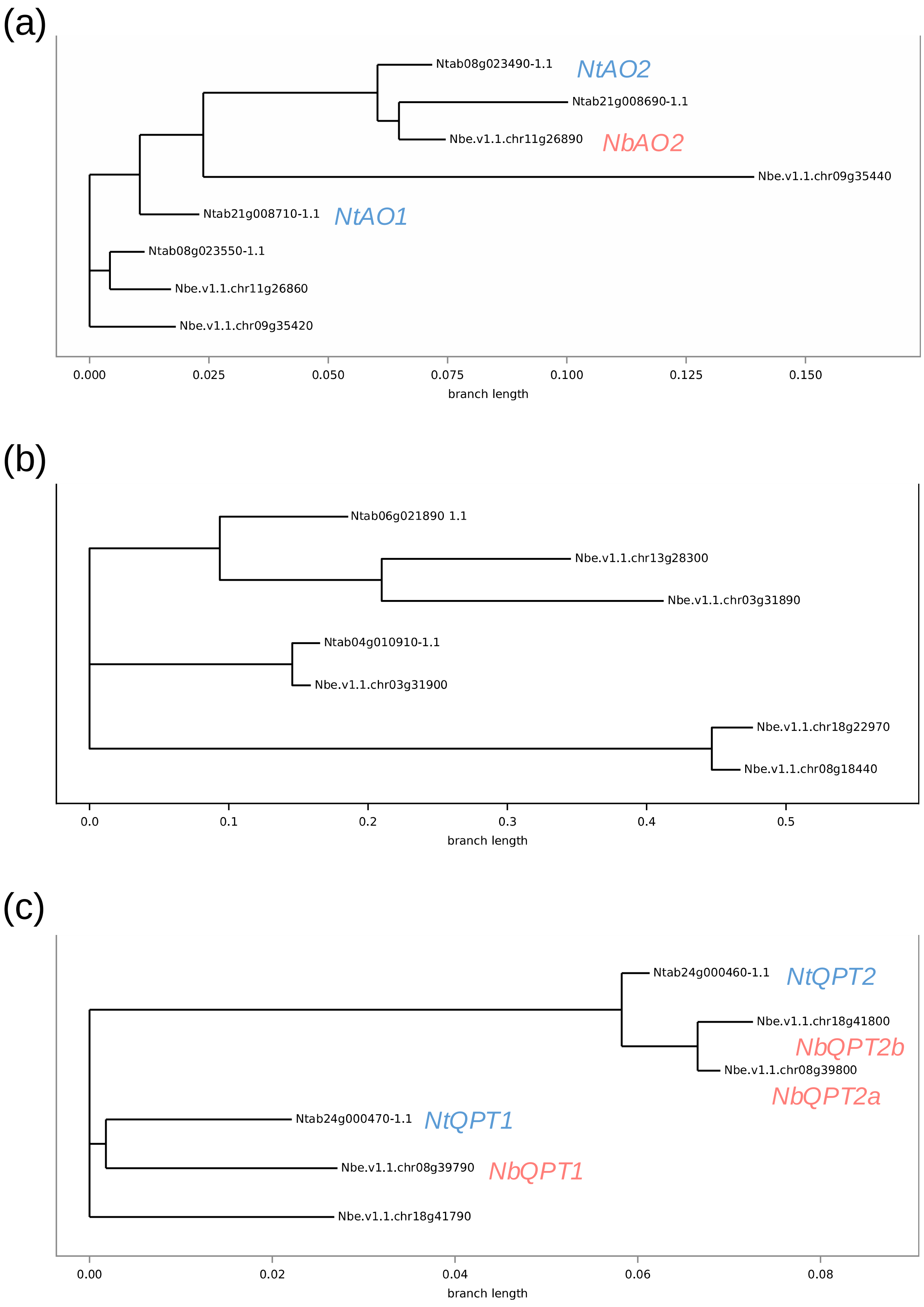

Supplementary Figure 6. Molecular phylogenetic trees of genes on the pyridine ring pathway

(a) Aspartate oxidase (AO). (b) Quinolinic acid synthase (QS). *Nbe.v1.1.chr03g31890.1* was excluded from the phylogenetic tree because it is extremely short compared to the other genes. (c) Quinolinic acid phosphoribosyltransferase (QPT).

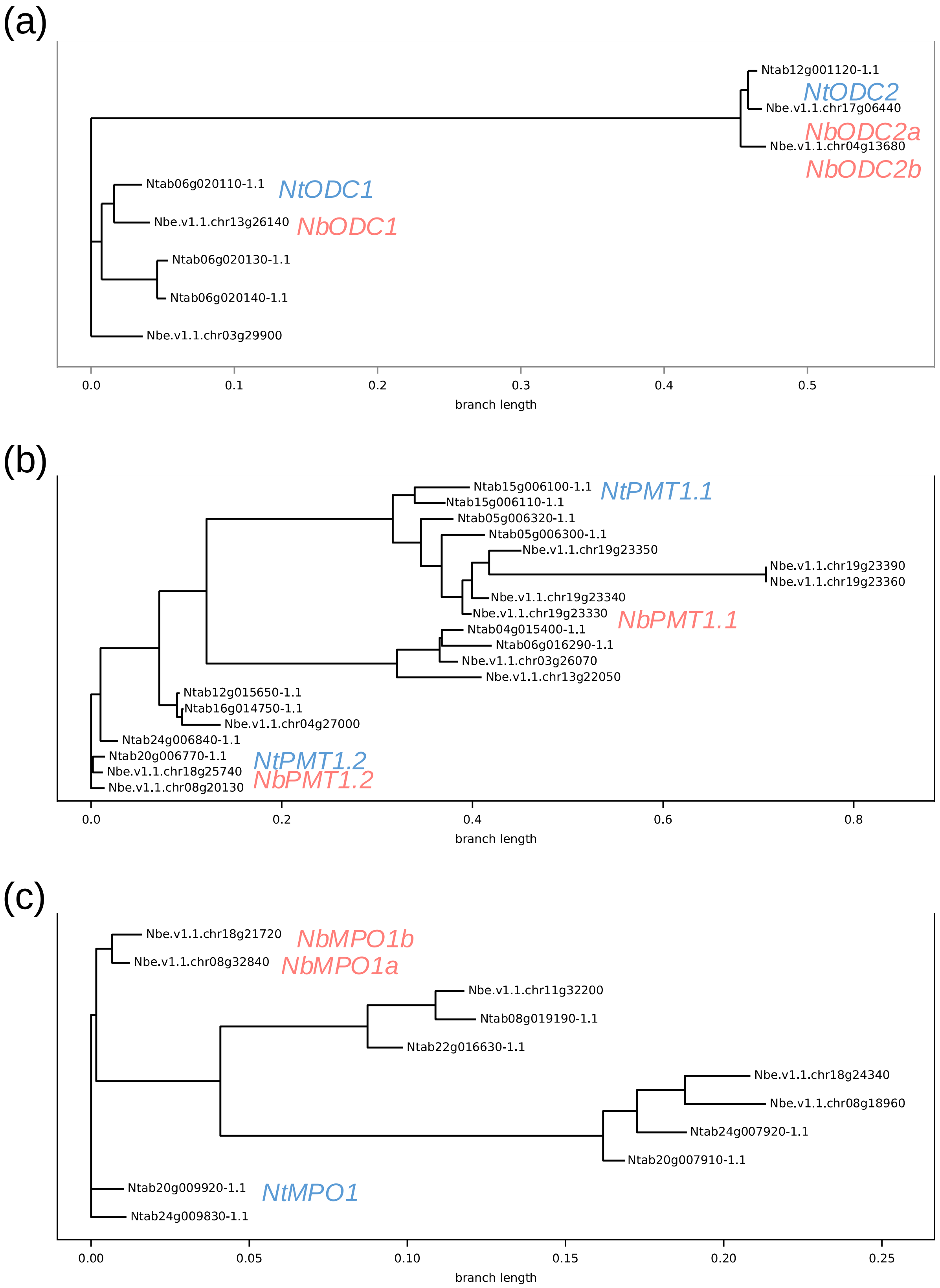

Supplementary Figure 7. Molecular phylogenetic trees of genes on the pyrrolidine ring pathway

(a) Ornithine decarboxylase (ODC). *Nbe.v1.1.chr17g19300.1* and *Nbe.v1.1.chr17g19310.1* were excluded from the phylogenetic tree because they are quite shorter than the other genes. (b) Putrescine *N*-methyltransferase (PMT). (c) *N*-methylptoresine oxidase (MPO).

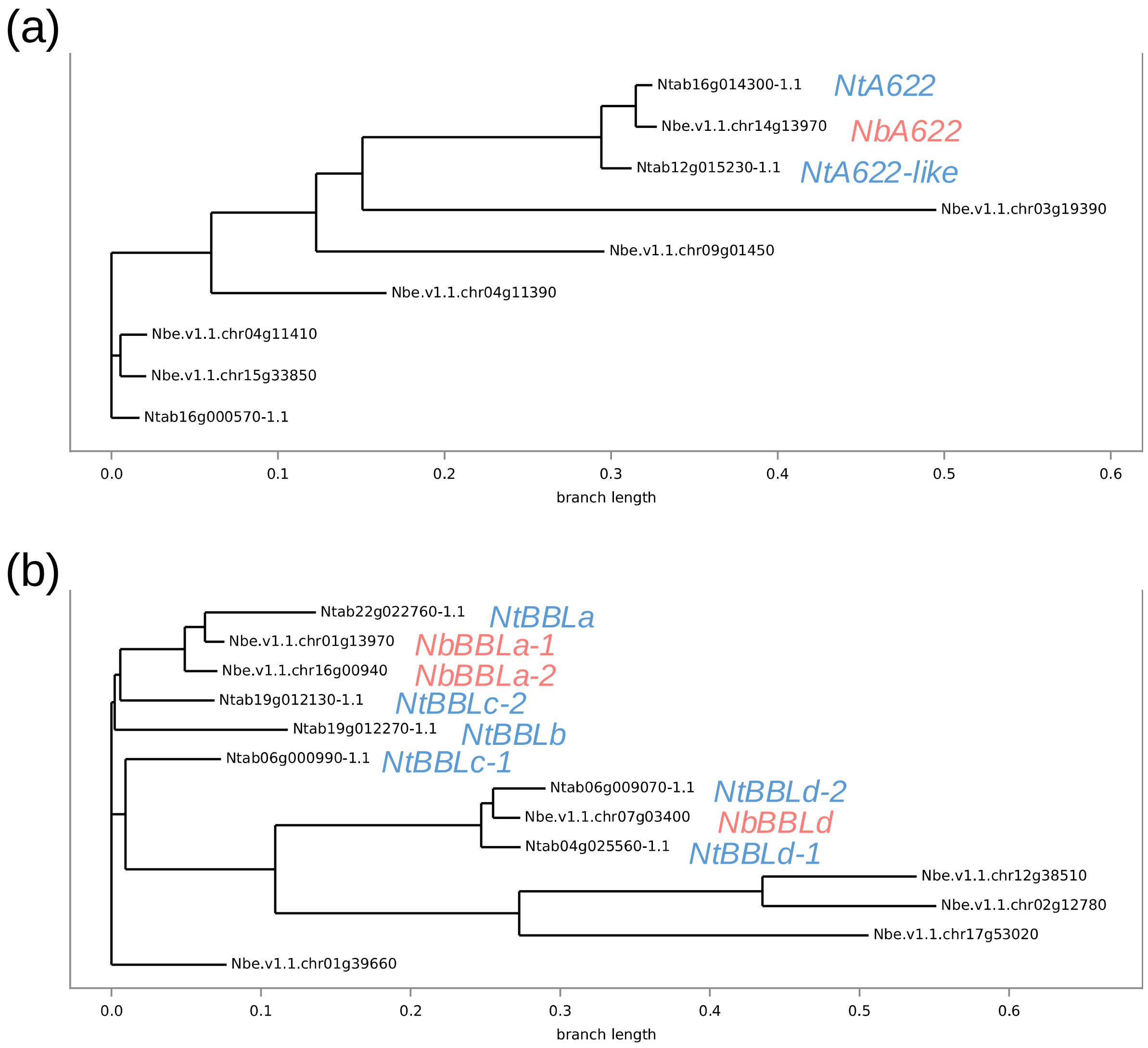

Supplementary Figure 8. Molecular phylogenetic trees of genes on the late bio-synthetic steps of pyridine alkaloids

(a) Phosphatidylinositol phosphate (PIP) family oxidoreductase A622. (b) Berberine bridge enzyme-like protein (BBL).

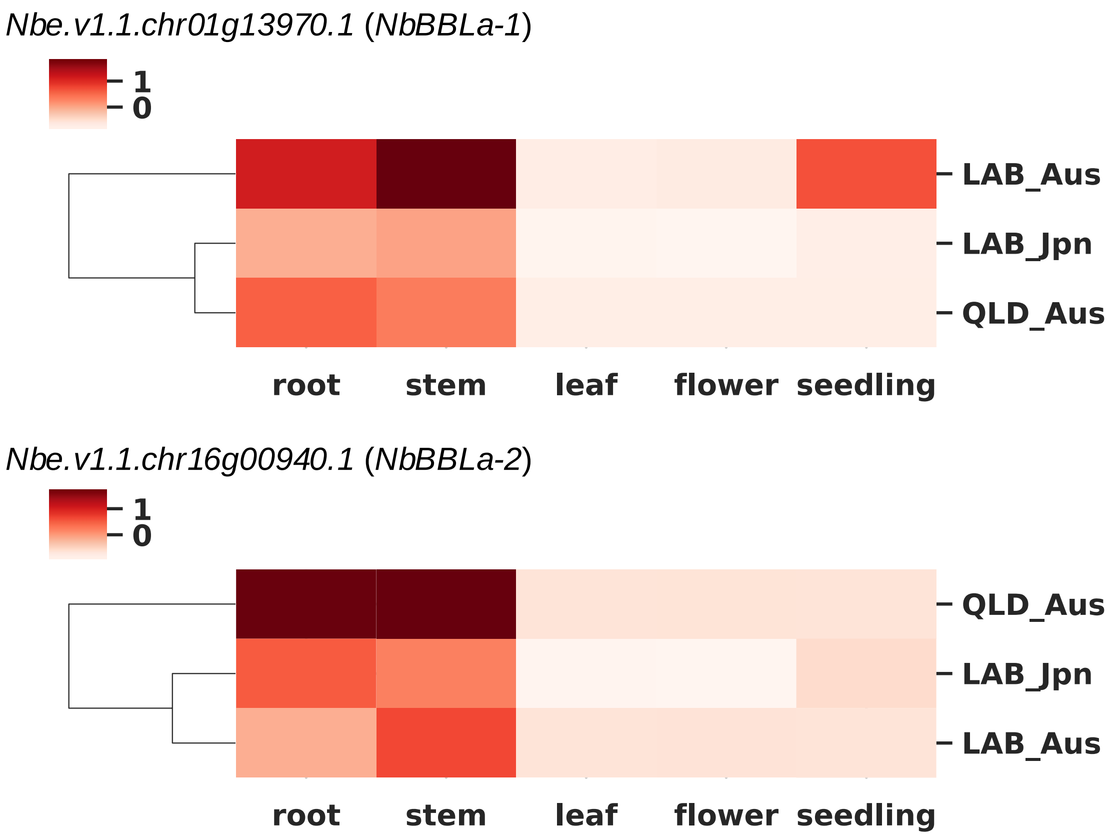

Supplementary Figure 9. Comparison of expression levels of *NbBBLa* genes in different stocks and different strains.

All RNA-seq data from the LAB strain used in the study in Japan (LAB_Jpn) and the LAB and QLD strains used in Australia (LAB_Aus and QLD_Aus, respectively) were mapped to Nbe.v1.1 as reference genome sequence. The expression of *NbBBLa-1* and *NbBBLa-2* were compared. ”leaf” in LAB_Jpn was the mean of “mature leaf”, “mature petiole”, “young leaf”, and “young petiole”, and “stem” was the mean of “stem 0-3”, “stem 3-6”, “stem 6-9”, and “stem 9-12”.

Biological Processes

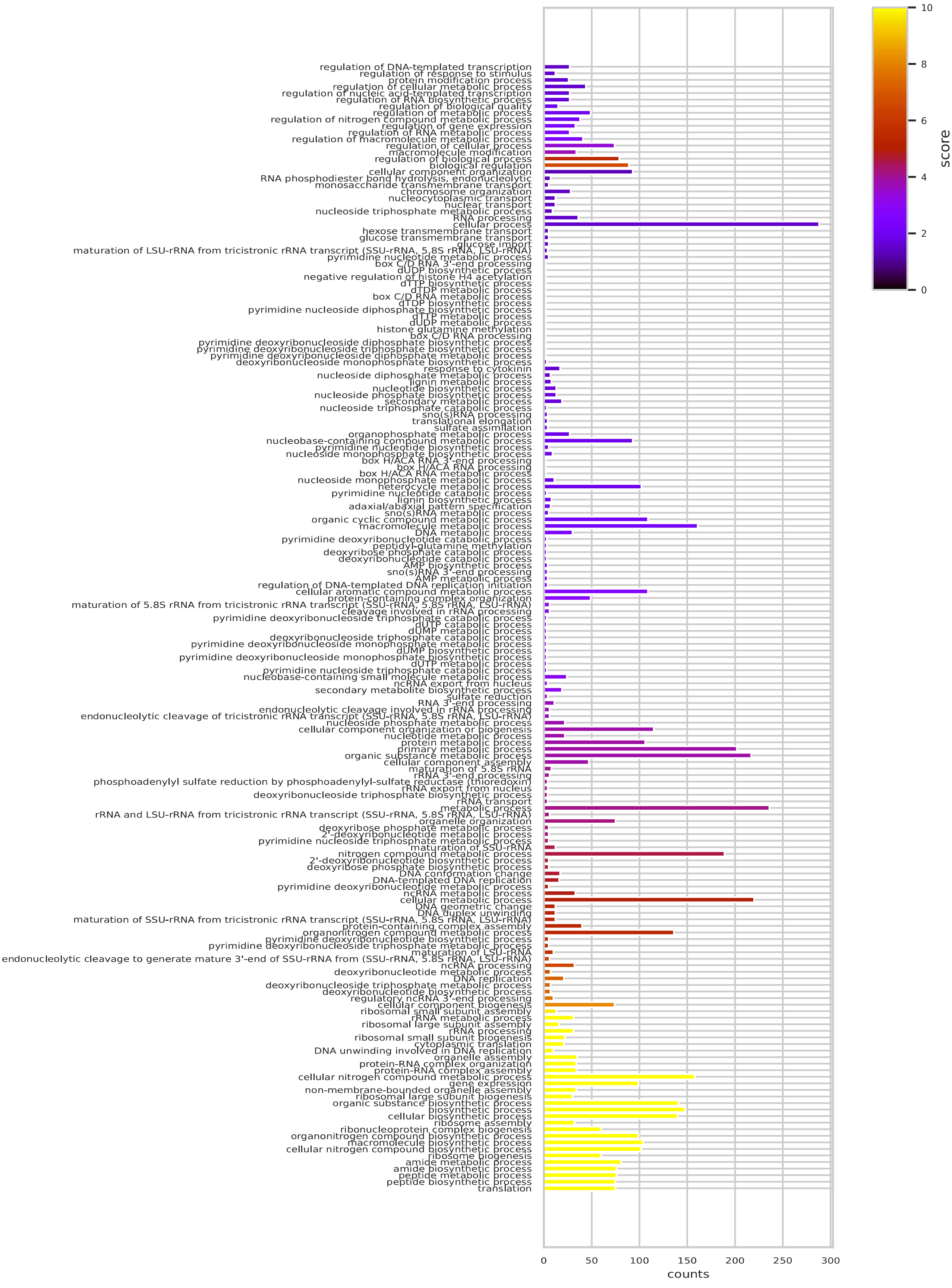

Supplementary Figure 10. Gene Ontology enrichment analysis of biological processes for up-regulated genes between buffer control and agroinfiltrated leaves.

Cellular Components

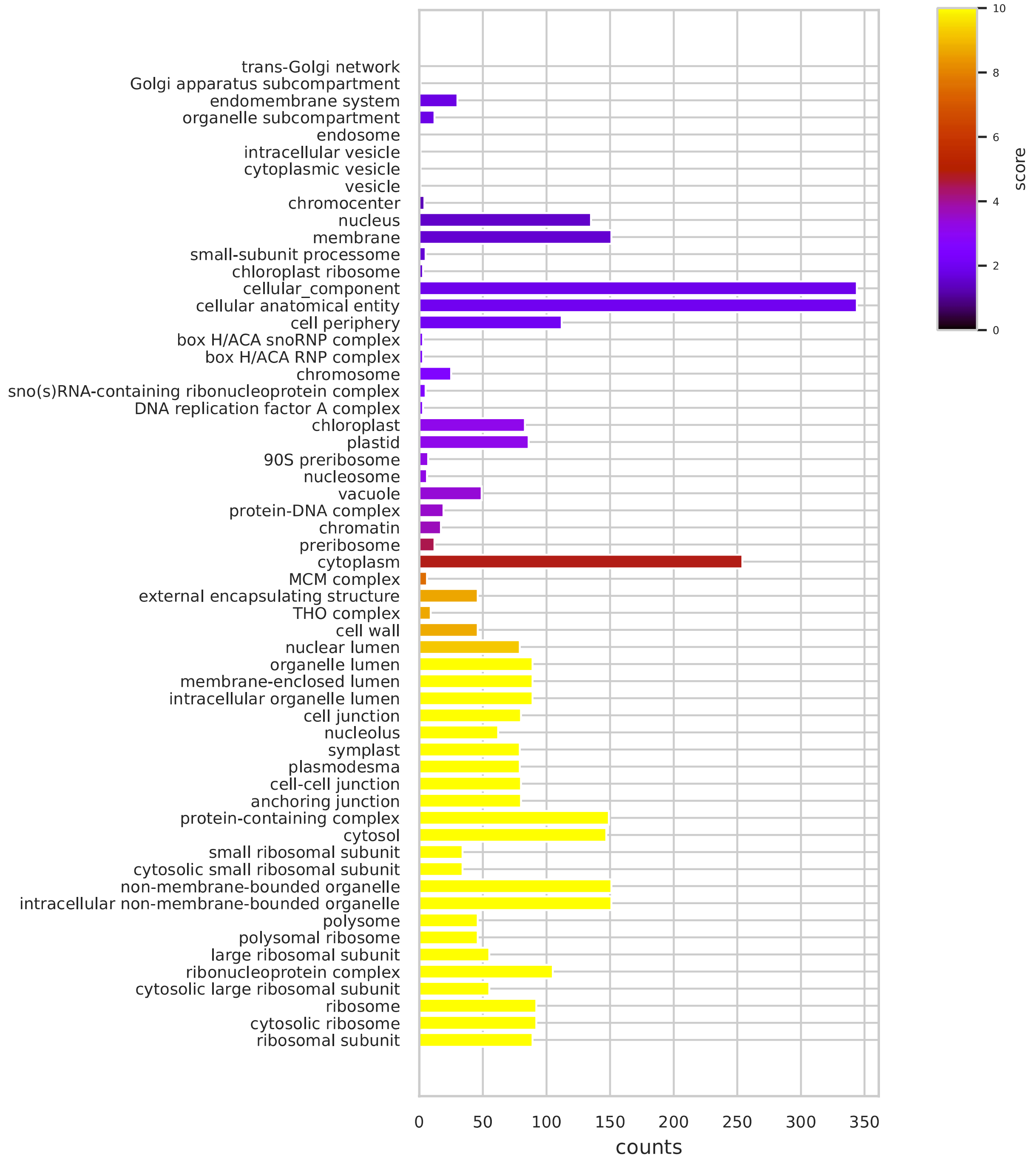

Supplementary Figure 11. Gene Ontology enrichment analysis of cellular components for up-regulated genes between buffer control and agroinfiltrated leaves.

Molecular Functions

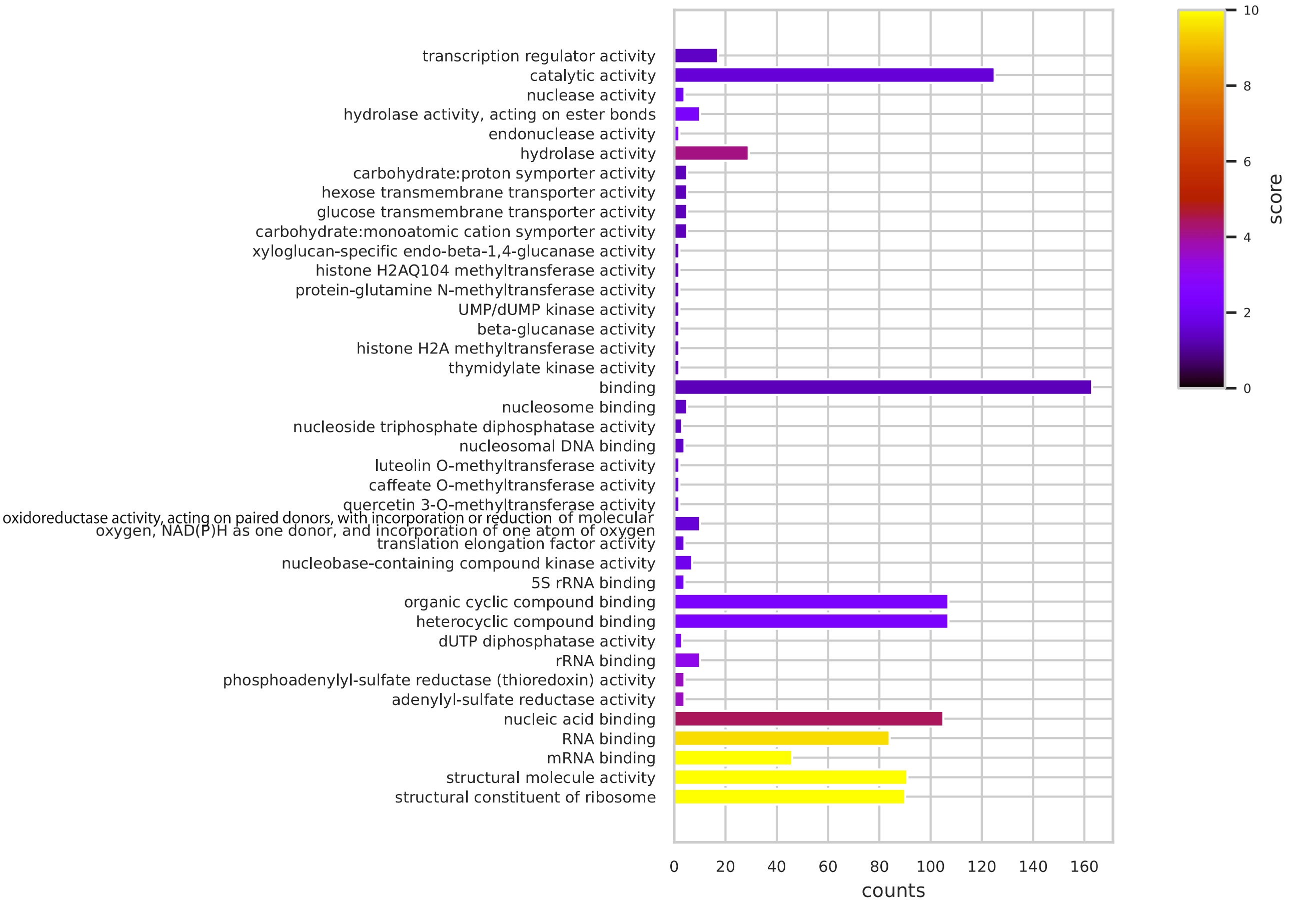

Supplementary Figure 12. Gene Ontology enrichment analysis of molecular functions for up-regulated genes between buffer control and agroinfiltrated leaves.
